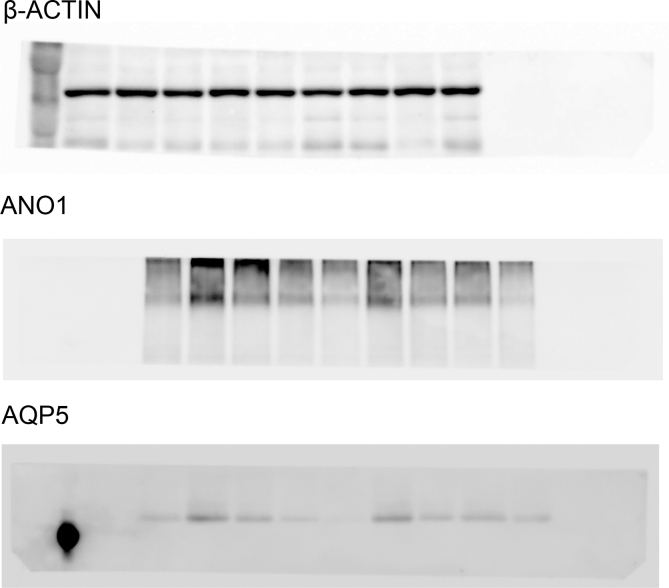# Light conditions affect rhythmic expression of aquaporin 5 and anoctamin 1 in rat submandibular glands

**DOI:** 10.1016/j.heliyon.2019.e02792

**Published:** 2019-11-14

**Authors:** Ryouichi Satou, Yoshiyuki Shibukawa, Maki Kimura, Naoki Sugihara

**Affiliations:** a Department of Epidemiology and Public Health, Tokyo Dental College, Chiyodaku, Tokyo 101-0061, Japan; b Department of Physiology, Tokyo Dental College, Tokyo, Chiyodaku, Tokyo 101-0061, Japan

**Keywords:** Cancer research, Physiology, Dentistry, Dental materials, Clock gene, Water metabolism, Aquaporin, Ion channels, Circadian rhythm

## Abstract

Circadian rhythms regulate various physiological functions and are, therefore, essential for health. Light helps regulate the master and peripheral clocks. The secretion rates of saliva and electrolytes follow a circadian rhythm as well. However, the relationship between the molecular mechanism of saliva water secretion and the peripheral circadian rhythm in salivary glands is not yet clear. The transmembrane proteins aquaporin5 (*Aqp5*) and anoctamin1 (*Ano1*) are essential for water transport in the submandibular glands (SGs). The purpose of this study was to reveal the effect of light conditioning on the peripheral clock in SGs. We examined temporal expression patterns among clock genes, *Aqp5* and *Ano1*, in rat SGs under light/dark (LD) and dark/dark (DD) conditions. We observed circadian rhythmic expression of *Bmal1*, *Per2*, *Cry1*, *Aqp5*, and *Ano1* mRNAs under both LD and DD conditions. The expression levels of *Aqp5* and *Ano1* peaked 6 h earlier under the DD condition than under the LD condition. Maintenance of the circadian rhythm of *Aqp5* and *Ano1* expression even under the DD condition indicates that *Aqp5* and *Ano1* may be controlled by clock genes; such genes are called clock-controlled genes (CCGs). Western blot analysis revealed the circadian oscillation and peak shift of AQP5 and ANO1expression under DD conditions. Clock genes may regulate the rhythmic expression of *Ano1* and *Aqp5* and may control osmic gradients in SGs.

## 1 Introduction

Circadian rhythms, which measure time on a scale of 24 h, regulate various physiological functions such as sleep cycle, blood pressure, hormone secretion, metabolism and salivary secretion in mammals [1, 2]. The rhythm is orchestrated by a master clock and several peripheral biological clocks. The master clock, which is located in the suprachiasmatic nucleus (SCN) of the hypothalamus, generates 24-hour circadian rhythms. In mammals, light resets the circadian timing of the master clock to synchronize with environmental conditions. Peripheral clocks in organs are regulated by a master clock [1]. Peripheral biological clocks are regulated independently by clock genes. The intracellular clock mechanism of the clock genes is based on transcriptional and translational feedback loops, which are called transcription translation oscillating loops (TTLs) [1, 3]. Among clock genes, aryl hydrocarbon receptor nuclear translocator-like protein 1 (*Arntl*/*Bmal1*), period circadian protein homolog 2 (*Per2*), cryptochrome circadian clock (*Cry*) and circadian locomotor output cycles kaput (*Clock*) are essential for TTLs. The key transcription factors CLOCK and BMAL1 form heterodimers which interact with the enhancer box (E-box) sequences in the promoters of *Per* and *Cry* genes, which drive the positive transcription of the TTLs [4]. The PER and CRY proteins interact, translocate into the nucleus and inhibit the activity of CLOCK-BMAL1 heterodimers, which promotes the transcriptional repression of the TTLs [5]. The master and the peripheral clocks in most tissues are controlled by this intracellular feedback loop. Dysregulation of clock gene expression results in diverse pathological conditions, such as sleep diseases, mental illness, cancers, metabolic syndromes, cardiovascular disorders and tooth development disorder [6, 7]. In recent years, the role of the circadian clock in the peripheral organs, such as heart, kidney and liver, has been investigated [4]. Multiple studies have suggested that the clock genes of peripheral clocks regulate physiological function in organs [4, 6, 8]. However, little is known about their roles in salivary glands.

The most potent entraining signal of circadian rhythm in mammals is light. Light induces a phase shift of the master clock in the SCN. Light entraining information reaches the SCN via the retinohypothalamic tract (RHT), which is the principal retinal pathway [1]. The SCN then relays this entraining information to peripheral clocks through endocrine signals and neural circuits. The phase of submaxillary *Per1* expression is controlled by light and food entrainment [9]. Light can synchronize peripheral clocks in mice through a *Syt10*-and *CamK2*-driven deletion of *Bmal1* in the SCN [10]. These studies suggest that light conditioning affects peripheral clocks and physiological function in organs.

Saliva plays an essential role in maintaining the integrity of the oral structures, in prevention of oral disease and in controlling oral infection. The importance of saliva in preventing the development of bacterial plaque formation [11]. The major salivary glands, submandibular glands (SGs) and the parotid and sublingual glands normally contribute over 90% of the total volume of unstimulated saliva [12]. The secretion of water and ions transport in SGs can be divided into two pathways: transcellular and paracellular transport pathways, which are driven by changes in water channel gating action and transmembrane osmosis [13]. Aquaporin 5 (AQP5) and Anoctamin 1 (ANO1) play an important role in water secretion and ion transport [14, 15, 16]. For driving the salivary secretions, AQPs regulate the transmembrane water movement in response to osmotic gradients. AQP5 is the major aquaporin expressed on the apical membrane of the intercalated ductal cells and acinar cells in SGs [15]. ANO1 is a transmembrane protein which functions as a Ca^2+^-activated chloride channel (CaCC). ANO1 are localized on the apical membrane and control the Cl^−^ efflux of apical in SGs. CaCCs are essential for the vectorial transport of electrolytes and water in the retina, airways, proximal kidney tubule epithelium, dorsal root ganglion sensory neurons and salivary glands [16, 17, 18].

The salivary flow rate and salivary substances such as Na^+^, Cl^−^, K^+^, HCO_3_^−^ and α-amylase secretion rate follows a circadian rhythm [2]. The unstimulated salivary flow rate is extremely low during sleep are known. Recent studies have shown a circadian rhythm of clock genes (deleted in esophageal cancer 1 [*Dec1*], *Dec2*, *Per1*, *Per2*, *Bmal1*, *Cry1*) and amylase 1 mRNA in submandibular glands (SGs) [19]. Clock proteins and *Bmal1* and *Per2* mRNAs localized in the mucous acini and striated ducts was determined by *in situ* hybridization [7]. These results suggest that clock genes play an important role in circadian oscillation of salivary secretion. However, rhythmic expression patterns of the clock genes, *Aqp5* and *Ano1* in SGs under different light condition remain to be investigated.

The purpose of this study was to reveal the effect of light conditioning on the peripheral clock in SGs. We examined temporal rhythmic expression patterns of the clock genes *Aqp5* and *Ano1* in rat SGs under light/dark (LD) and dark/dark (DD) conditions.

## 2 Materials and methods

### 2.1 Animals and ethical approval

Six-week-old male Wistar rats (Charles River Laboratories Japan, Inc., Tsukuba, Japan) were used for this study. Only male rats were chosen to avoid the effect of sex-related hormonal differences. Rats were maintained for 2 weeks on a light/dark (LD)-cycle of 12 h light and 12 h dark prior to all experiments, and food and water were available ad libitum. To determine the effects of light exposure, we kept the rats in constant darkness under a dark/dark cycle (DD) for 48 h before sampling. All experiments were performed in conformity with zeitgeber time (ZT) with 8:00 set as ZT0. This study was approved by the Ethics Committee of Tokyo Dental College after the review by Institutional Animal Care and Use Committee (Permission number: 290901, 300901) and carried out the Guidelines for the Treatment of Experimental Animals at Tokyo Dental College. All animals were treated in accordance with the Council of the Physiological Society of Japan and the American Physiological Society. We isolated the glands before lights on at the transition states DD to LD (in dark room). At DD to LD, Rats were anesthetized and the SGs were extracted in the dark to avoid the effect of light stimulation.

### 2.2 RNA isolation and real-time semi-quantitative RT-PCR (sqPCR)

Total RNA from submandibular glands (SGs) at ZT0, 6, 12, 18, 24, 30, 36, 42 and 48 h were isolated with RNAiso Plus (TaKaRa Bio, Shiga, Japan). RNA concentration and quality were determined using a spectrophotometer NanoDrop-2000 (Thermo scientific, Waltham, MA, USA). We used the same quantity of total RNA (50 ng) for all series of sqPCR analyses. Total RNA from SGs was subjected to real-time semi-quantitative RT-PCR analysis (Thermal Cycler Dice, TaKaRa Bio). Expression level of the internal reference gene (*β-actin*) was measured using One Step SYBR® PrimeScript™ RT-PCR Kit II (Perfect Real Time, TaKaRa Bio). Probes labeled with 6-carboxyfluorescein (6-FAM) was used. The primers used were gene-specific primers for *β-actin*, *Bmal1*, *Per2*, *Clock*, *Cry1*, *Ano1* and *Aqp5* (Table 1). The comparative Ct method (2^−ΔΔCt^, where Ct denotes cycle threshold) was used for sqRT-PCR analysis. We assessed the candidate gene expression relative to that of *β-actin* using the Thermal Cycler Dice real time system software version 5.11.

**Table 1:** Primer sequences for sqPCR.

Gene Name		5′-sequence-3′	GenBank Number
*β-actin*	Forward	GGAGATTACTGCCCTGGCTCCTA	NM_031144.3
	Reverse	GACTCATCGTACTCCTGCTTGCTG	
*Bmal1*	Forward	TTCATGAACCCGTGGACCAA	NM_024362.2
	Reverse	CCCTGGAATGCCTGGAACA	
*Per2*	Forward	TCTCAGAGTTTGTGCGATGATTTG	NM_031678.1
	Reverse	CACTGGGTGAAGGTACGTTTGG	
*Clock*	Forward	ACACAGCCAGCGATGTCTCAA	NM_021856.1
	Reverse	CATGGCTCCTAACTGAGCTGAAAG	
*Cry1*	Forward	CGGCGACCTATGGATCAGTTG	NM_198750.2
	Reverse	TCCCAGCATTGATGCTCCAG	
*Ano1*	Forward	TCAAAGGCCGGTTTGTTGGTCG	NM_001107564.1
	Reverse	GGCGAAGGGTTCGAGGTTGAAG	
*Aqp5*	Forward	GCCGTCAATGCGCTGAACAAC	NM_012779.1
	Reverse	CATGGAACAGCCGGTGAAGTAGATC	

*Bmal1*, aryl hydrocarbon receptor nuclear translocator-like protein 1; *Per2*, period 2; *Clock*, circadian locomotor output cycles kaput; *Cry1*, cryptochrome circadian clock 1; *Ano1*, Anoctamin 1; *Aqp5*, Aquaporin 5; GenBank Number; the Accession number of NIH genetic sequence database.

### 2.3 Western blot analysis

The SG tissue were harvested at CT0, 6, 12, 18, 24, 30, 36, 42 and 48, and homogenized in ice cold radioimmunoprecipitation assay (RIPA) lysis buffer (188–02453, Fujifilm-wako Corp., Osaka, Japan). Protein samples concentration were calculated using the DC protein assay kit (Bio-Rad, Richmond, CA) based on the Lowry method. For each sample, 10 μg protein was electrophoresed on 10% SDS-PAGE gel, transferred to Immobilon-P Transfer Membrane (PVDF, Millipore, Burlington, Massachusetts, USA) membrane and analyzed using the Mini Trans-Blot® Transfer Cell (#1703930JA, Bio-Rad, Richmond, CA). PVDF membrane were blocked with 5% skimmed milk for 1h, and probed overnight at 4 °C with anti-ANO1 (1:500, ab53212; Abcam, Cambridge, UK), anti-Aquaporin 5 (1:5000, ab78486; Abcam, Cambridge, UK) and anti-β ACTIN (1:10000, GTX110564; GeneTex, Alton Pkwy, US). Horse-radish peroxidase (HRP)-conjugated polyclonal goat anti-rabbit immunoglobulins (1:1000, P0448; Dako, California, US) was used for 1 h at room temperature. Protein bands were visualized with the ECL chemiluminescence WB Detection Reagents (GE Health Care, Little Chalfont, UK), and documented using the Image Quant LAS-4000 (GE Health Care). Quantification of bands were performed by using Image Quant TL 7.0 software (GE Health Care).

### 2.4 Statistical analysis

All sqPCR data are displayed as the mean ± SD. Circadian rhythms during 48-h periods were statistically analyzed by one-way analysis of variance (ANOVA) and p < 0.05 were considered significant differences, with the Bonferroni test for post hoc comparisons when significance was determined by analysis of variance. All western blot results were represented as mean ± SD from five independent experiments. P-values were calculated by one-way ANOVA and significant differences observed at p < 0.05. The Bonferroni test for post hoc comparisons was performed and p < 0.01 were considered significant differences. Rhythmicity was analyzed by CircWave version 1.4 (Oster et al., 2006) and the significance (p < 0.05) of rhythmicity was evaluated at a 95% confidence level (α = 0.05).

## 3 Results

### 3.1 Circadian rhythm of clock genes in SGs under LD and DD conditions

**Fig. 1:**
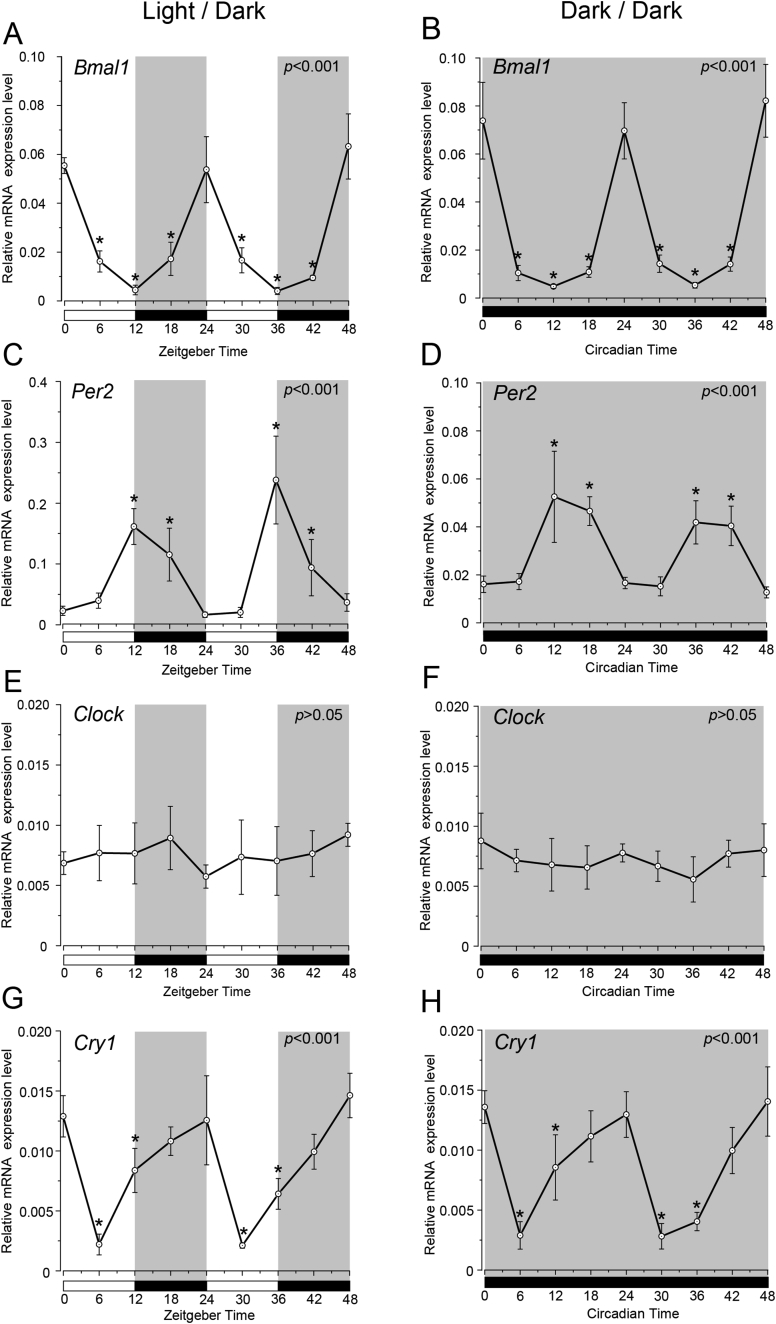
Temporal expression profiles of clock genes in the submandibular glands under LD and DD conditions. Gene expression levels of (A) *Bmal1*, (C) *Per2*, (E) *Clock* and (G) *Cry1* at 6 h intervals in zeitgeber time (ZT: lights on at ZT0 and ZT24; lights off at ZT12 and ZT36) under the LD condition. Gene expression levels of (B) *Bmal1*, (D) *Per2*, (F) *Clock* and (H) *Cry1* at 6 h intervals in circadian time (CT: continuous dark condition) under the DD condition. The horizontal white and black bars indicate light and dark phases (shown by gray), respectively. The mRNA levels are displayed as the mean ± SD of five replicates per time point (n = 5). P-values were calculated by one-way ANOVA and results were considered significant at p < 0.05. The Bonferroni test for post hoc comparisons was performed and p < 0.05 were considered significant differences compare to CT0 (or ZT0) are indicated as ‘*’. Rhythmicity was determined using CircWave (p < 0.05) at a 95% confidence level (α = 0.05). *Bmal1*, *Per2* and *Cry1* showed rhythmic mRNA expression patterns in both LD and DD conditions.

We observed rhythmic mRNA expression patterns of *Bmal1*, *Per2*, *Clock* and *Cry1* in SGs under both DD and LD conditions. We examined temporal relative expression of the clock genes mRNA in the SGs every 6 h from ZT0 to ZT48 (five experiments, with 45 rats in total; Fig. 1). Relative expression levels of *Bmal1* mRNA were significantly higher at ZT0, ZT24 and ZT48 and were lower at ZT12 and ZT36 in the LD condition (Fig. 1A). *Bmal1* mRNA showed significant rhythmic expression both LD and DD conditions (one-way ANOVA, p < 0.01). The peak times of *Bmal1* expression in LD and DD overlapped (Fig. 1B). Temporal relative expression of *Per2* mRNA showed significantly higher expression at ZT12 and ZT36 and lower expression at ZT0, ZT24 and ZT48 (Fig. 1C) in the LD condition. Relative expression levels of *Per2* mRNA showed similar results in the DD condition (Fig. 1D). *Bmal1* expression showed antiphase with the expression pattern of *Per2* with a 12 h phase difference.

*Clock* mRNA did not show significant rhythmic expressions (one-way ANOVA, p > 0.05) and a clear phase variation could not be observed in its expression peaks both LD and DD conditions (Fig. 1E, F). *Cry1* mRNA showed significant upregulation at ZT24 and ZT48 and lower expression at ZT6 and ZT30 (Fig. 1G). The phase of expression of *Cry1* mRNAs deviated by 12 h from the phase of *Per2* expression peaks in LD and DD conditions (Fig. 1C, D, G, H). The peak-to-peak periods of *Bmal1*, *Per2* and *Cry1* were maintained for 24 h, and peak times were consistent between LD and DD conditions (Fig. 1A, B, C, D, G, H). The expression levels of *Bmal1*, *Per2* and *Cry1* were considered rhythmic by CircWave in both LD and DD conditions (Fig. 1).

### 3.2 Circadian rhythm of *Aqp5* and *Ano1* in SGs under LD and DD conditions

**Fig. 2:**
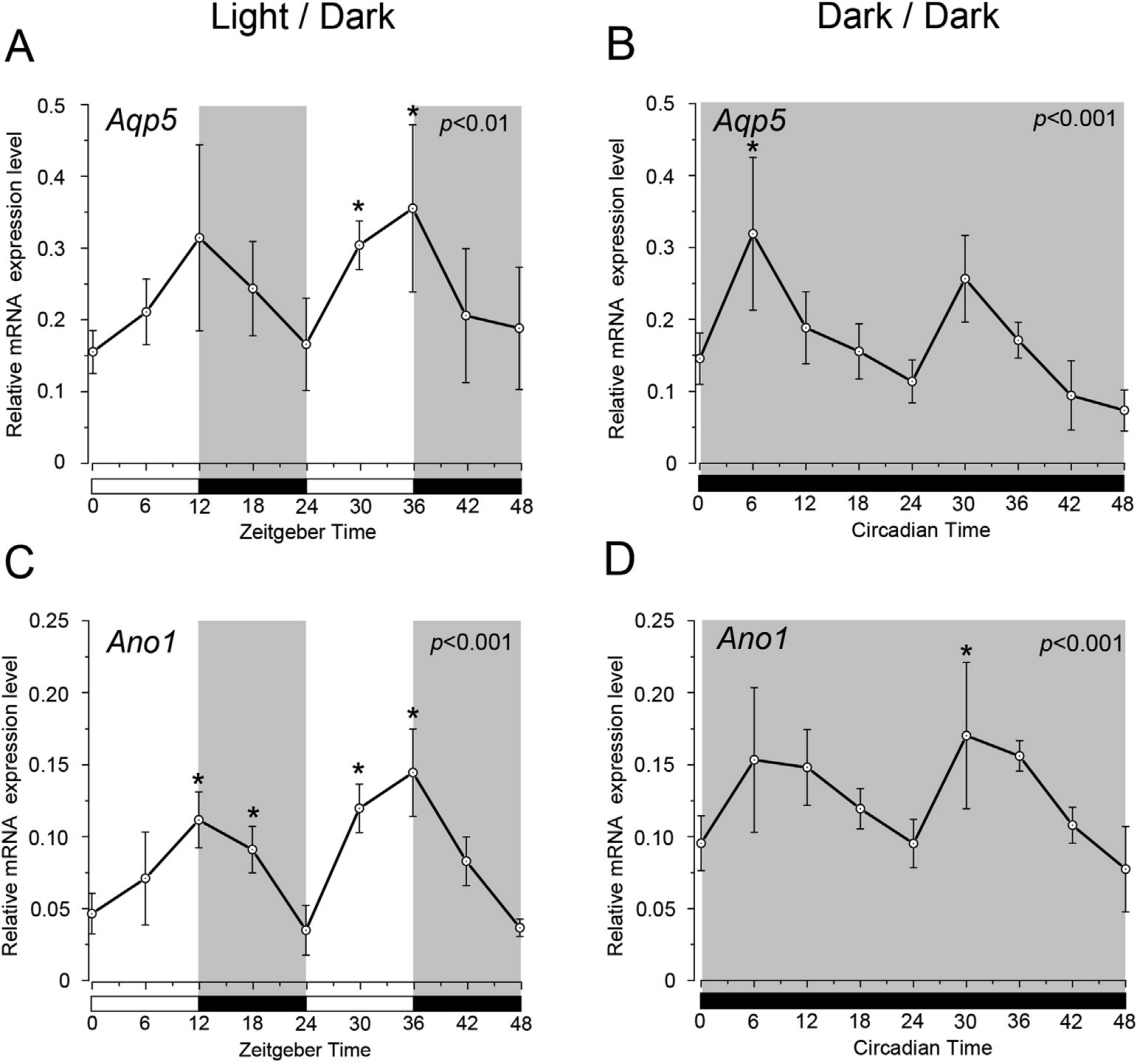
Temporal expression profiles of *Aqp5* and *Ano1* in submandibular glands under LD and DD conditions. Gene expression levels of (A) *Aqp5* and (C) *Ano1* at 6 h intervals in zeitgeber time (ZT) under the LD condition. Gene expression levels of (B) *Aqp5* and (D) *Ano1* at 6 h intervals in circadian time (CT) under the DD condition. The horizontal white and black bars indicate light and dark phases (shown by gray), respectively. The mRNA levels are displayed as the mean ± SD of five replicates per time-point (n = 5). P-values were calculated by one-way ANOVA and results were considered significant at p < 0.05. The Bonferroni test for post hoc comparisons was performed and p < 0.05 were considered significant differences compare to CT0 (or ZT0) are indicated as ‘*’. Rhythmicity was determined using CircWave (p < 0.05) at a 95% confidence level (α = 0.05). *Aqp5* and *Ano1* showed rhythmic mRNA expression patterns in both LD and DD conditions. The peak times of *Aqp5* and *Ano1* expressions under the DD condition occurred 6 h earlier than that under the LD condition.

We observed temporal mRNA expression profiles of *Aqp5* and *Ano1* in the saliva (Fig. 2). Temporal relative expression profiles of *Aqp5* and *Ano1* mRNAs in SGs were examined every 6 h from ZT0 to ZT48 (five experiments using 45 rats in total; Fig. 2). The relative expression levels of *Aqp5* mRNA were higher at ZT12 and ZT36, whereas they were lower at ZT0, ZT24, and ZT48 under the LD condition (Bonferroni test, p < 0.05) (Fig. 2A). *Aqp5* mRNA showed significant rhythmic expression under both LD and DD conditions (one-way ANOVA, p < 0.01). The expression pattern of *Aqp5* mRNA differed under the LD and DD conditions (Fig. 2A, B). *Aqp5* mRNA expression was upregulated at ZT6 and ZT30, but was significantly downregulated at ZT0, ZT24, and ZT48 under the DD condition (Bonferroni test, p < 0.05) (Fig. 2B). The peak time of *Aqp5* expression under the DD condition occurred 6 h earlier than that under the LD condition (Fig. 2A, B). The same phase was observed for *Ano1* mRNA expression, with ZT12 and ZT36 showing the highest, and ZT0, ZT24, and ZT48 showing the lowest expression under the LD condition (Fig. 2C). *Ano1* showed rhythmicity, with a significantly higher expression observed at ZT6 and ZT30 and a lower expression observed at ZT0, ZT24, and ZT48 (Fig. 2D). The peak times of *Ano1* expression under the DD condition occurred 6 h earlier than those under the LD condition (Fig. 2C, D). The expression levels of *Aqp5* and *Ano1* were considered rhythmic through CircWave analysis under both LD and DD conditions (Fig. 2).

### 3.3 Circadian rhythm profiles of AQP5 and ANO1 protein under DD conditions

**Fig. 3:**
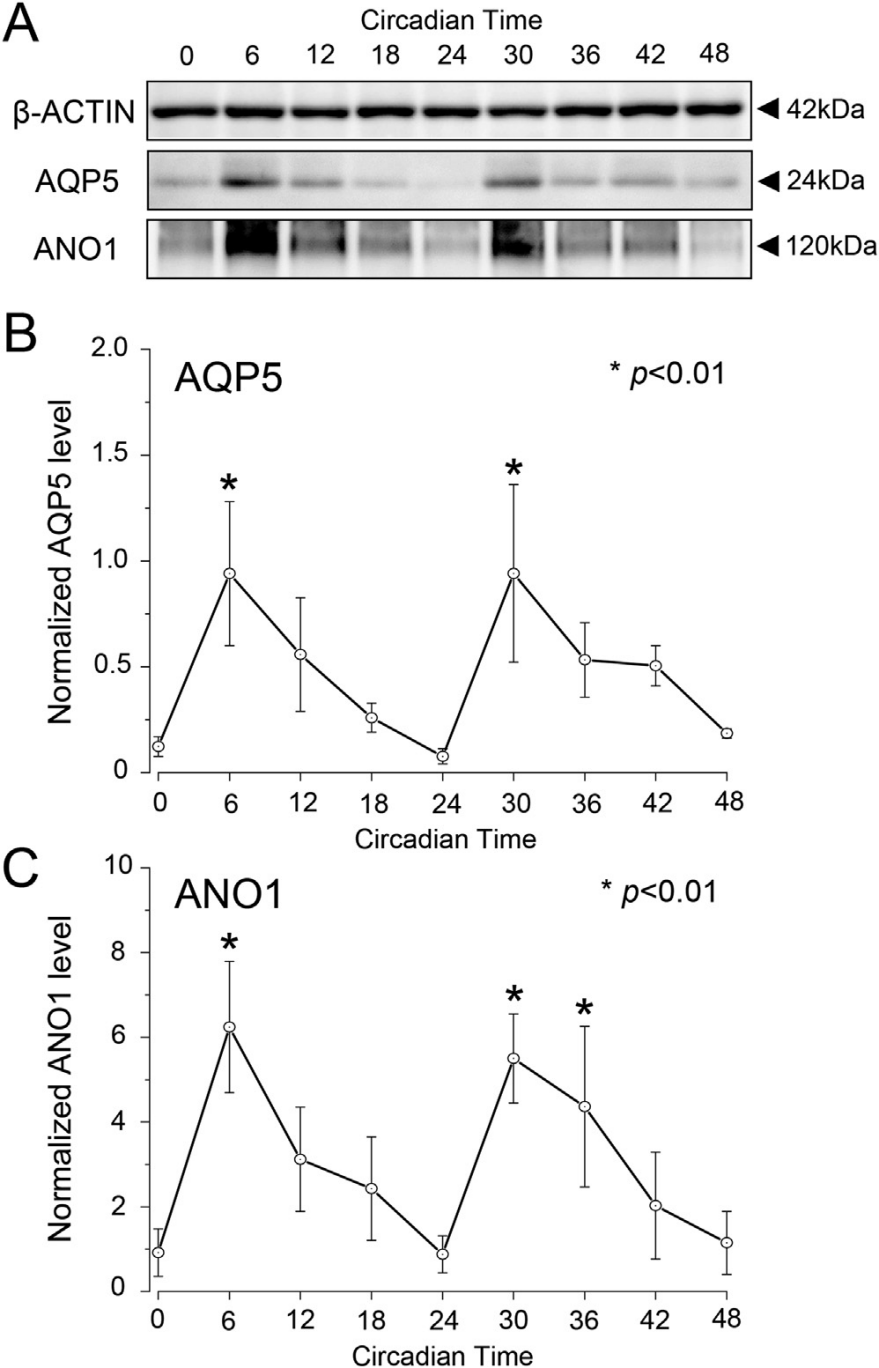
Circadian oscillation of AQP5 and ANO1 expression in the rat SGs under DD conditions by Western blot analysis. Protein expression characterization (A) and relative levels of AQP5 (B) and ANO1 (C) at 6 h intervals in CT. The expression level was normalized with ß-ACTIN. For each time point, SG samples were loaded at 10 μg total protein per lane. Markers at band indicate relative mass (in kDa). The relative levels are displayed as the mean ± SD of five replicates per time-point (n = 5). P-values were calculated by one-way ANOVA and results were considered significant at p < 0.05. The Bonferroni test for post hoc comparisons was performed and p < 0.01 were considered significant differences compare to CT0 are indicated as ‘*’. Non-adjusted and sliced images are shown in supplementary Fig. 1.

Western blot analysis revealed the circadian oscillation of AQP5 and ANO1 expression in the rat SGs under DD conditions. The expression of ß-ACTIN single band was shown in 42 kDa, and did not exhibit any circadian patterning during 48 h (Fig. 3A). A single band (24 kDa) was detected for AQP5 (Fig. 3A). AQP5 expression was normalized with ß-ACTIN, a constitutively expressed internal control, every 6 h for a 48 h period. The expression levels of AQP5 protein were higher at CT6 and CT30, whereas they were lower at CT0, CT24, and CT48 (Bonferroni test, p < 0.01) (Figs. 3A and 3B). A wide single band (110–120 kDa) was detected for ANO1 (Fig. 3A). The circadian expression of ANO1 showed significant oscillation patterns peaking at CT6 and CT30 (Bonferroni test, p < 0.01) (Figs. 3A and 3C).

## 4 Discussion

### 4.1 Clock genes in SGs showed circadian rhythm under LD and DD conditions

We demonstrated that *Bmal1*, *Per2*, *Clock*, and *Cry1* show rhythmic circadian expression in SGs under LD and DD conditions (Fig. 1). The phases and peaks of *Bmal1 and Per2* expression profiles showed opposite rhythms, which were shifted by 12 h and repeated every 24 h. The *Cry1* expression peaks were shifted compared to those of *Per2* and *Bmal1*. It was reported that the temporal expression profile of the *Clock* in the SCN does not show any circadian rhythm. This is in agreement with our results. The phases and peaks in the expression profiles of genes examined by us were similar to those in other organs and SCN [5, 20]. Our results under the LD condition are consistent with those of previous studies [19, 21]. Clock gene mRNA expression phases are maintained in SGs under the DD condition. These results suggest that SGs have peripheral clock mechanisms with negative feedback loops. The 48 h in continuous darkness did not change clock gene mRNA expression phases in SGs. Our results suggest that the phases of clock genes in SGs might be not rapidly affected by the light condition.

### 4.2 *Aqp5* and *Ano1* in SGs showed circadian rhythm under LD and DD conditions

*Aqp5* and *Ano1* showed rhythmic circadian expression similar to that of *Per2* under the LD condition (Fig. 2A, C). Expressions of *Aqp5* and *Ano1* followed a circadian rhythm pattern in SGs under the DD condition (Fig. 2B, D). The temporal expression pattern of *Aqp5* showed similar patterns and peak times as that of *Ano1* under both the LD and DD conditions (Fig. 2). Our results suggest that exposure to light shifts the peak expression time of *Ano1* and *Aqp5* in the rat SGs. However, we did not conduct a flash exposure experiment in this study under DD condition, therefore it is unclear whether light stimulation can cause a shift in the peripheral clock of the SGs. We showed that AQP5 and ANO1 protein expression displayed rhythmic circadian oscillations. There was no time-lag between the peak time of protein and mRNA expression under DD condition (Figs. 2 and 3). The result indicated that *Aqp5* and *Ano1* mRNA translated promptly without any delay, and *Aqp5* and *Ano1* Peak shift was maintained not only in mRNA but also in protein (Fig. 3).

Upregulation of *Aqp5* and *Ano1* mRNA expression drive changes in transmembrane osmosis and water channel gating in SGs [16]. In *Aqp5* knock-out mice, more than 60% saliva production was reduced and the tight junction proteins and water permeability was decreased expression of compared to the wild-type [16, 22, 23]. The intracellular Ca^2+^ concentration, which additionally showed circadian rhythm were controlled by the activation of ANO1 [24]. *Ano1* disruption by siRNA transfection in mice significantly reduced the salivary flow rate induced by muscarinic-cholinergic stimulation [16]. These results suggest that the circadian rhythm in water secretion may be controlled with water permeability, which is influenced by the circadian oscillation of *Aqp5* and *Ano1* expressions. In the nocturnal period, Rats increase intake of water and food (ZT12−ZT24). The temporal expression peaks of *Aqp5* and *Ano1* correlated with their feeding and drinking behavior [25, 26]. In previous our studies, we examined DNA sequences to confirm the relationship between clock genes and *Aqp5* and *Ano1* expression. The enhancer box (E-box) binding sequence, which acts as the binding site of the BMAL1-CLOCK heterodimer to the promoter region was found on rat *Aqp5* and *Ano1* [27]. The existence of an E-box in the promoter region and the maintenance of rhythmic expression cycles under the DD condition are characteristics of clock-controlled genes (CCGs) [28]. Our results suggest that *Aqp5* and *Ano1* are putative CCGs and target gene of the BMAL1-CLOCK heterodimer in SGs. Phase shifts of *Aqp5* and *Ano1* under the DD condition indicated that light condition may be one of the synchronization factors in SGs.

### 4.3 The pathway of light condition affects physiological functions in rat SGs

There are two main factors involved in the synchronization of peripheral clocks to environment conditions: light and feeding. For example, timed food uptake can primarily reset the liver clock and thereby regulate liver metabolism [6]. In the present study, all experiments were carried out with food and water available ad libitum. Therefore, light was considered to be the main factor affecting synchronization. Light from the retina reaches the SCN through the RHT and entrains the master clock. Then, master clock transmits timing information to peripheral clocks along neuronal and endocrine pathways (pathway1). We observed a differential expression in peak time of *Aqp5* and *Ano1* mRNAs between the LD and DD conditions. However, no time shifts in clock gene peak expression were observed (Figs. 1 and 2). These results are inconsistent with entrain pathways through SCN (pathway1) because clock genes in the peripheral clock were not shifted under the DD condition. Therefore, it is presumed that there exist additional pathways through which peak time are synchronized independently of the SCN. Several reviews on the synchronization of the peripheral clocks have recently been published describing that light can reach peripheral clocks via several routes. Additional pathways exist through which peripheral clocks are synchronized independently of the SCN clock (pathway2) [10,29]. There were shift in only part of the waveform of *Aqp5* and *Ano1*. In addition to the mechanism that shifts the entire waveform like pathway1, there may be pathway2 in the SGs that provides quick adjustments to the partial ambient light environment.

In conclusion, we show circadian rhythmic expression of *Bmal1*, *Per2*, *Cry1*, *Aqp5* and *Ano1* mRNAs in LD and DD conditions. We show different circadian rhythmic expressions of *Aqp5* and *Ano1* between the LD and DD conditions. Maintaining the rhythm of *Aqp5* and *Ano1* even in the absence of light stimulus indicates that *Aqp5* and *Ano1* may be controlled by clock genes as CCGs. Clock genes may regulate the rhythmic expression of *Ano1* and *Aqp5* mRNA and may control osmic gradients in SGs.

## Declarations

### Author contribution statement

Ryouichi Satou: Conceived and designed the experiments; Performed the experiments; Analyzed and interpreted the data; Contributed reagents, materials, analysis tools or data; Wrote the paper.

Maki Kimura: Performed the experiments; Contributed reagents, materials, analysis tools or data.

Yoshiyuki Shibukawa, Naoki Sugihara: Conceived and designed the experiments.

### Funding statement

This work was supported by JSPS KAKENHI Grant Number JP19K18953.

### Competing interest statement

The authors declare no conflict of interest.

### Additional information

Data associated with this study has been deposited at GenBank under the following accession numbers: β-actin NM_031144.3, Bmal1 NM_024362.2, Per2 NM_031678.1, Clock NM_021856.1, Cry1 NM_198750.2, Ano1 NM_001107564.1, Aqp5 NM_012779.1.

No additional information is available for this paper.

## Figures and Tables

**Supplementary Fig1:**